# CTHNet: a network for wheat ear counting with local-global features fusion based on hybrid architecture

**DOI:** 10.3389/fpls.2024.1425131

**Published:** 2024-07-02

**Authors:** Qingqing Hong, Wei Liu, Yue Zhu, Tianyu Ren, Changrong Shi, Zhixin Lu, Yunqin Yang, Ruiting Deng, Jing Qian, Changwei Tan

**Affiliations:** ^1^ Jiangsu Key Laboratory of Crop Genetics and Physiology/Jiangsu Key Laboratory of Crop Cultivation and Physiology, Agricultural College of Yangzhou University, Yangzhou, China; ^2^ Jiangsu Co-Innovation Center for Modern Production Technology of Grain Crops/Joint International Research Laboratory of Agriculture and Agri-Product Safety of the Ministry of Education of China/Jiangsu Province Engineering Research Center of Knowledge Management and Intelligent Service, College of Information Engineer, Yangzhou University, Yangzhou, China

**Keywords:** deep learning, transformer, wheat ear counting, density detection, feature fusion

## Abstract

Accurate wheat ear counting is one of the key indicators for wheat phenotyping. Convolutional neural network (CNN) algorithms for counting wheat have evolved into sophisticated tools, however because of the limitations of sensory fields, CNN is unable to simulate global context information, which has an impact on counting performance. In this study, we present a hybrid attention network (CTHNet) for wheat ear counting from RGB images that combines local features and global context information. On the one hand, to extract multi-scale local features, a convolutional neural network is built using the Cross Stage Partial framework. On the other hand, to acquire better global context information, tokenized image patches from convolutional neural network feature maps are encoded as input sequences using Pyramid Pooling Transformer. Then, the feature fusion module merges the local features with the global context information to significantly enhance the feature representation. The Global Wheat Head Detection Dataset and Wheat Ear Detection Dataset are used to assess the proposed model. There were 3.40 and 5.21 average absolute errors, respectively. The performance of the proposed model was significantly better than previous studies.

## 1 Introduction

For the management and phenotyping of wheat production, an accurate wheat ear count is essential. Traditional techniques of counting wheat mostly rely on manual counting, which is prone to human subjectivity, leading to inaccurate identification and omission of wheat ears and low efficiency (Fernandez-Gallego et al., 2018; Zhou et al., 2018; Sadeghi-Tehran et al., 2019). To increase the efficiency and accuracy of counting wheat, effective automatic counting techniques must be created (Patrício and Rieder, 2018).

Deep learning techniques have been frequently used in wheat counting tasks because of their strong computational and feature extraction capabilities. The current methods used for wheat ear counting mainly include three methods: image segmentation, object detection, and density estimation. For high-density wheat ear counting in complex field backgrounds, it mainly relies on object detection methods and density estimation methods. The former uses deep learning algorithms to pinpoint and identify specific wheat ears.

In order to automatically count wheat ears, (Cao et al., 2020) used the deep learning model efficientdt-d3 for target detection while counting the number of targets based on image processing and deep learning. (Li et al., 2022) used a two-stage detection method to explore the use of Faster R-CNN model to detect the number of ears per unit area of wheat based on RGB images and applied it to genotype analysis. The model successfully localized the target genotypes and successfully predicted the quantity of wheat ears with an average accuracy of 86.7%. An enhanced Yolov4 model based on the Convolutional Block Attention Module (CBAM) (Woo et al., 2018) was also proposed by (Yang et al., 2021). The model can adaptively learn and select significant spatial and channel information in the feature map, efficiently eliminate background interference, and improve the network’s ability to extract features by introducing spatial and channel attention methods. (Sun et al., 2022) suggested an enhanced wheat ear counting network (WHCnet) to address the issue that wheat ears are frequently overlooked. Utilizing both the original information and the underlying knowledge of the features, adaptive pooling of original information using Augmented Feature Pyramid Network (AugFPN) (Guo et al., 2020) enhances the effectiveness of wheat ear identification. (Wen et al., 2022) introduced a weighted Bidirectional Feature Pyramid Network (BiFPN) (Tan et al., 2020) into the feature pyramid network of RetinaNet (Lin et al., 2017) to fuse multiscale features. Meanwhile, focus loss and attention modules were added to train a RetinaNet based on several optimizations, which successfully achieved effective detection and counting of wheat ears. The wheat counting algorithm based on object detection mentioned earlier uses convolutional operations to obtain local features of the image, and obtains more comprehensive and rich feature maps through multi-scale and attention mechanisms, without considering the global contextual information of the image. In addition, in order to achieve optimal performance, the high performance of object detection algorithms largely rely on clear images and accurate labeling. However, it takes a lot of time and effort to classify small objects using bounding boxes, particularly for high-density wheat images.

In recent years, scientists have given the density map-based wheat counting model a lot of attention. For counting and localization, the method uses point-labeled data, which requires less effort than box labeling. Convolutional neural networks are used to create high-quality density maps that accurately depict the distribution of wheat. The density maps may be summed to calculate the number of wheat ears, and they are ideally suited to tiny and dense situations. (Pound et al., 2017) built an hourglass network for multi-task learning based on an encoder/decoder structure to simultaneously localize ear characteristics and categorize awn phenotypes in wheat grown in greenhouses. (Khaki et al., 2022) proposed a new method for wheat ear counting called WheatNet. Accelerated the detection rate by using MobileN-etV2 (Sandler et al., 2018) as the primary feature extractor with less parameters. For wheat ear counts and localization, the model uses two parallel subnets that complement one another and boost prediction precision. (Ma et al., 2022) proposed a transfer learning method of the ground-based fully convolutional network. Filter pyramid blocks and dilated convolution are combined to successfully address the issue of counting performance degradation brought on by a reduction in ground resolution. In order to locate and count wheat ear points, (Zaji et al., 2022) developed a hybrid Unet (Ronneberger et al., 2015) structure combining a point-labeled dataset and a constant density graph generation algorithm, considerably enhancing the accuracy of wheat counting. (Xiong et al., 2019) built the tasselnetv2 local regression network for counting wheat by including a contextual information extraction module to the local patches. Without expanding the model’s capacity, the accuracy of the counting was increased. (Lu and Cao, 2020) implemented a fast version of TasselNetV2, TasselNetV2+, by splitting TasselNetV2 into an encoder, a counter, and a normalizer based on a novel framework view of TasselNetV2. This fast version improves the speed by an order of magnitude compared to TasselNetV2, while maintaining the same level of counting accuracy. The wheat ear counting method based on density estimation performs better in dealing with complex backgrounds and high-density wheat ears, as it focuses on the overall distribution of wheat ears rather than the precise position of individual wheat ears. Compared to object detection methods, density estimation methods typically have higher computational efficiency and lower costs. The above research indicates that the use of object detection methods and density estimation methods in agriculture can significantly improve the automation level of agricultural production and produce better results. The current methods are mainly based on convolutional neural networks because of their powerful local feature extraction capabilities, which perform better than the traditional methods (Liu et al., 2019) based on artificial feature extraction. However, because the convolutional operation is limited to the convolutional kernel acceptance domain, it is unable to comprehend an image’s global information. In most computer vision tasks, this global information and long-range feature dependence are crucial components. Because the self-attention mechanism can handle a long range of feature dependencies and has a significant advantage in extracting global context information, Transformers based on the self-attention mechanism have been widely used in vision tasks in recent years (Carion et al., 2020).

The aim of this study is to increase the diversity of features by combining local features and global context information. We introduce a new network for wheat ear counting with local-global features fusion based on hybrid architecture. The main contributions of this paper can be summarized as follows: (1) We design a new model that combines a Cross Stage Partial (CSP) (Wang et al., 2020) for extracting local features with a Pyramid Pooling Transformer (P2T) (Wu et al., 2023) for capturing global context information to acquire features at multiple scales. Our method has stronger feature extraction capability and significantly improves the wheat counting performance. (2) We propose a feature fusion module for fusing image local features and global context information. The module can effectively integrate local features and global context information. (3) We design a new hybrid loss function that combines the wheat counting loss and the attention loss to train the hybrid network and improve the counting performance of the model. (4) Our proposed CTHNet method is compared with the counting methods proposed in previous research. Experimental results on two commonly used datasets, Global Wheat Head Detection Datasets (GWHD) (David et al., 2021) and Wheat Ears Detection Dataset (WEDD) (Madec et al., 2019), show that our method achieves better performance in the wheat counting task.

The structure of the paper is as follows: the second part describes the selection of the two public wheat datasets, the data preprocessing process, and a general introduction to the proposed model. The third part includes the evaluation metrics, experimental setup, performance comparison, and a discussion of the method of this paper and other comparative methods. Finally, the fourth part summarizes the main findings of the study and looks at possible future research directions.

## 2 Materials and methods

### 2.1 Data set and processing

#### 2.1.1 Data set

**Figure 1:**
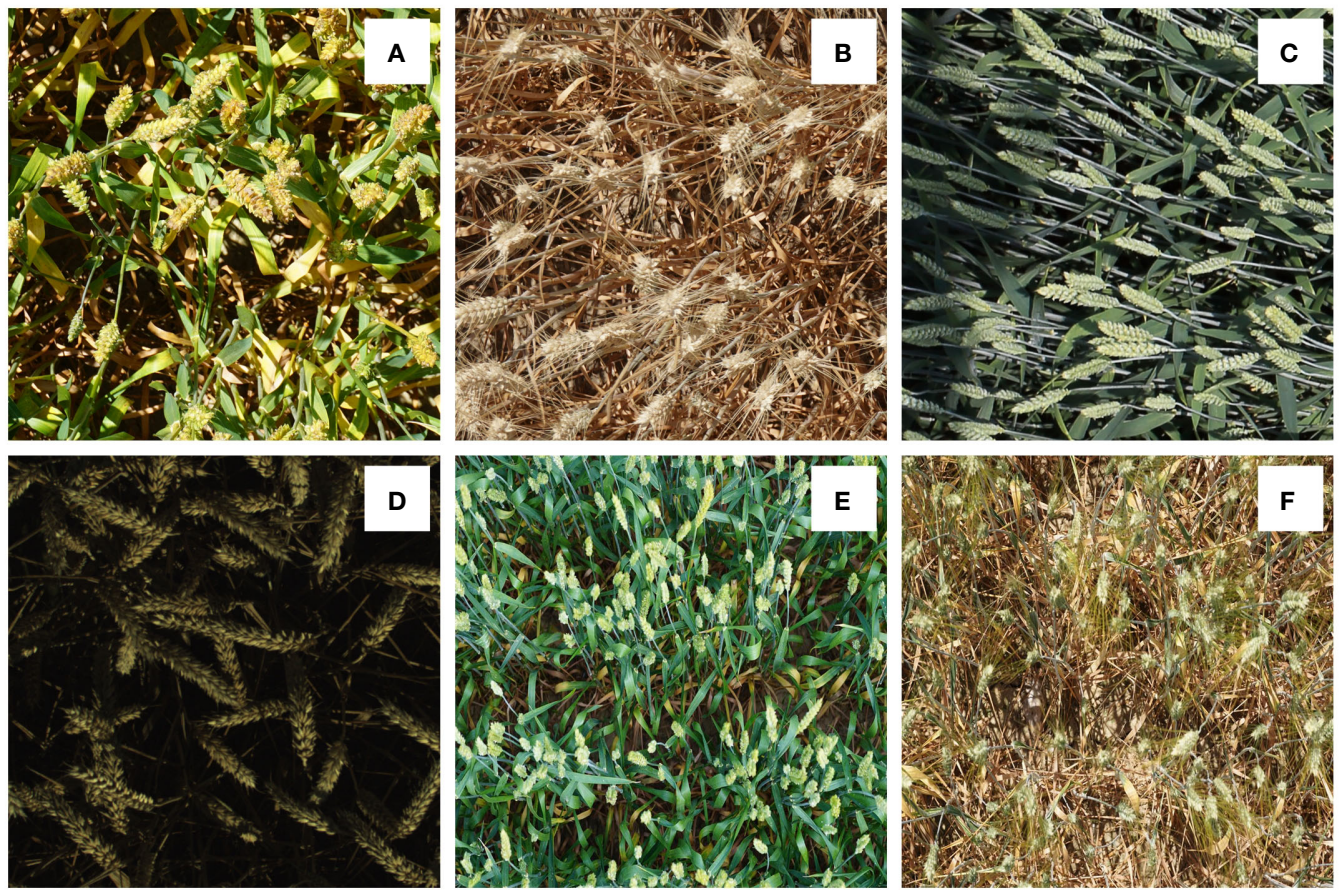
Wheat dataset diversity: **(A)** diversity in genotype, **(B)** maturity, **(C)** head orientation, **(D)** lighting, **(E, F)** density.

In this work, we utilized two publicly available wheat ear datasets: (1) GWHD, (2) WEDD. Example images from two datasets were shown in **Figure 1**. In addition, the details of each dataset are as shown in **Table 1**.

**Table 1:** The statistics of dataset used in this study.

Data	Dataset	Number of Image	Resolution	Min	Max	Avg	Total
GWHD	Training	3574	1024x1024	4	129	45.67	163,237
	Test	1440	1024x1024	4	179	30.79	44,331
WEDD	Training	165	6000x4000	82	245	133.85	22,085
	Test	71	6000x4000	106	176	134.32	9,537

Min, Max, Avg, and Total denote the minimum, maximum, average, and total number of annotated wheat heads, respectively.

The GWHD is a large-scale image dataset specially used to detect wheat ears. The data set aims to promote the research and development of computer vision and machine learning in the field of agriculture. As shown in **Figure 1**, this dataset contains a large number of high-resolution wheat field images from all over the world. The images cover different geographical areas, different planting conditions, and different wheat varieties. The shape and color of wheat ears vary greatly in the dataset, and there is overlap between wheat ears. Accurate localization and counting of wheat ears is challenging due to the influence of many factors. Each image is professionally marked with the exact position of the wheat ears, which is represented by a bounding box, providing the accurate position and size information of the wheat ears. The images contain a total of 147,722 wheat ears samples, with an average of 43 wheat ears in each image.

The second dataset is from the public dataset WEDD provided by (Madec et al., 2019). This dataset was collected on a wheat field phenotype analysis platform using a Sony ILCE-6000 digital camera. It contains 236 high-resolution wheat images (6000 × 4000 pixels) with a total of 31,622 wheat ears. The number of ears in each image varies from 80 to 240. **Table 1** presents the comprehensive statistics of the training and test data sets. The image  in the dataset is reshaped to 1024×1024 pixels in size and include as many wheat ear samples as possible to reduce hardware pressure and unify labeling requirements.

#### 2.1.2 Ground truth density map

This study uses a method based on density map to calculate the number of wheat ears, which needs to use point annotation to generate a real density map. Therefore, we need to use point annotation instead of box annotation to re label the image of dataset. We first use the bounding box annotation provided by dataset to calculate the centroid of each bounding box, so as to obtain the dataset of point annotation.

Let $P=\left\{p_{1},p_{2},\ldots \ldots ,p_{n}\right\}$ denote the annotation set of N wheat ears. Each ear can be represented by a delta function $\delta \left(x-p_{i}\right)$ . Therefore, we can represent the ground truth of a wheat ear image with N annotations as follows:


$$H\left(x\right)=\sum_{i=1}^{N}\delta \left(x-p_{i}\right)$$

Where $p_{i}$ is the position of the i th wheat ear and x is the all-zero matrix of the same size as the labeled image. The function $\delta \left(x-p_{i}\right)$ purpose is to set the $p_{i}$ position in the matrix x to 1.

A Gaussian function $G_{\sigma }\left(x\right)$ is used to smooth the discrete density map generated by H(x) and transform it into a continuous function to generate the ground truth density map F(x) :


$$F\left(x\right)=\sum_{i=1}^{N}\delta \left(x-p_{i}\right)*G_{\sigma }\left(x\right)$$

Where σ represents the standard deviation, which is set to a constant in the density maps we generate. The resulting ground truth density map has the property that the sum on the density map is the same as the total number of small wheat ears in the image.

Based on the generated density map, proceed to compute the attention map using the Gaussian kernel as follows:


$$\text{Z}=\text{F}\left(\text{x}\right)*{\text{G}}_{\text{σ}}\;\left(\text{x}\right)$$

$$\forall x\in Z,A\left(x\right)=\{\begin{array}{c} 0,\;\;x<\text{th}\\ 1,\;\;x\geq \text{th} \end{array}$$

Where th is the threshold set in our experiment, which is set to 0.001 (Zhu et al., 2019).

#### 2.1.3 Data augmentation

To obtain high-quality datasets, additional data augmentation methods such as random cropping are added to increase the variability of the training set. Considering the distribution density of small wheat ears in wheat images, we chose an image size of 512 pixels for random cropping.

**Figure 2:**
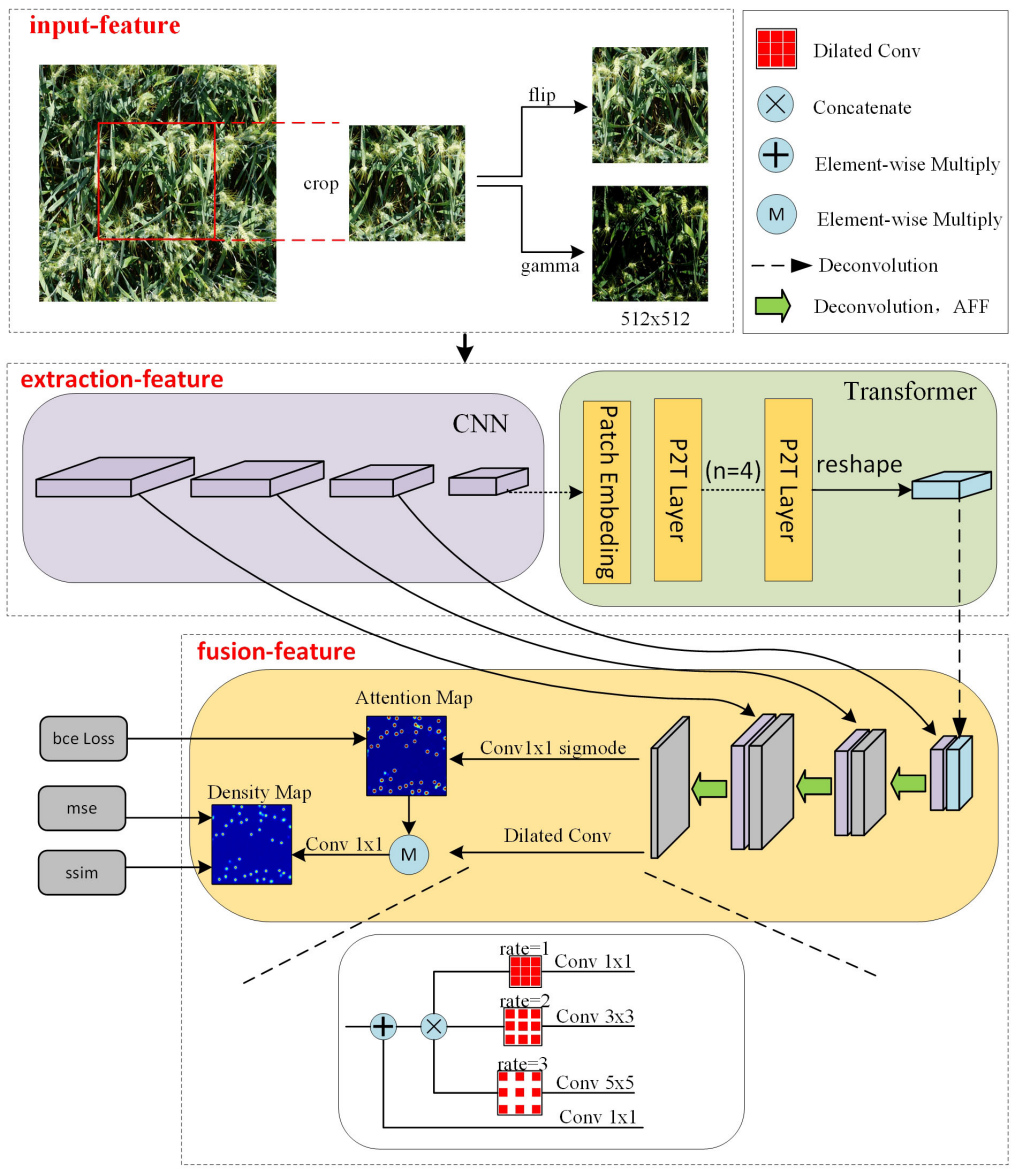
The overall framework of hybrid network includes feature extraction module, multi-scale feature aggregation module.

In this study, multiple enhancement techniques were randomly applied to the images. Since all augmentations are done randomly, the model cannot see exactly similar input images during training. This means that the model needs more time to train thoroughly to improve the generalization performance of the model. The different data augmentation techniques used in training are shown in **Figure 2**. After cropping, the enhancement process includes the hue change of the image and the flip of the picture, and these enhancements are all done randomly on the image with a probability of 0.5. This means that there is a 50% chance of applying each enhancement method to the original image.

### 2.2 Hybrid network model

As shown in **Figure 2**, we constructed a network structure in which the input images for the training process are obtained by randomly clipping the original images. Taking the input image size of 512 × 512 pixels as an example. Firstly, local features are extracted through convolution network, using four convolutional kernels with of 2 and channel dimensions of 64, 256, 512and 512 to obtain multiscale feature maps with sizes of 256 × 256 pixels, 128 × 128 pixels, 64 × 64 pixels and 32 × 32 pixels respectively. The large-scale feature map is derived from the shallow network, which has high resolution and good detail feature representation ability. On the contrary, the small-scale feature map is extracted from the deep network and contains more semantic information, but the resolution is low and the representation ability of spatial details is weak. Patch embedding is applied to extract 1×1 patch from the minimum scale feature map extracted by CNN, capturing the global contextual information of the feature maps while keeping the resolution constant. This is helps to consider local details and global information in image processing and improves the understanding of image content. Then, multi-scale feature fusion is carried out through the attention mechanism to output the prediction density map. When generating the density map, the dilated convolution layers with different expansion rates are superimposed in parallel to make the predicted density map contain more spatial information and expand the perception domain. Taking the RGB image as input, three features are input, and the features are increased to 512 in the feature extraction stage, and then the features are reduced to two by the feature fusion, one for the loss calculation of the density map and one for the loss calculation of the attention map.

#### 2.2.1 Feature extraction

The hybrid network is mainly composed of two parts, which realize the task of wheat ear density counting. These two parts are feature extraction and feature fusion. In the feature extraction, we combine the self-attention mechanism with CSP module to build a new network structure. This special design structure can effectively extract local features and global context. Through CNN convolution and global attention mechanism, the local details and global context information of the image can be paid attention to simultaneously in the process of feature extraction. In the process of feature fusion, we enhance the detection ability of the hybrid attention network for wheat at different scales by fusing multi-scale local and global features.

In our proposed backbone network, CSP was used to construct a CNN network to extract the local features of the image. CSP structure is a convolutional neural network structure commonly used to construct the backbone network. By dividing the feature map of the base layer into two parts, the gradient flow propagates through different network paths, finally the feature maps of the beginning and end stages of the network are integrated. It can effectively improve the learning ability and training speed of convolutional neural network.

**Figure 3:**
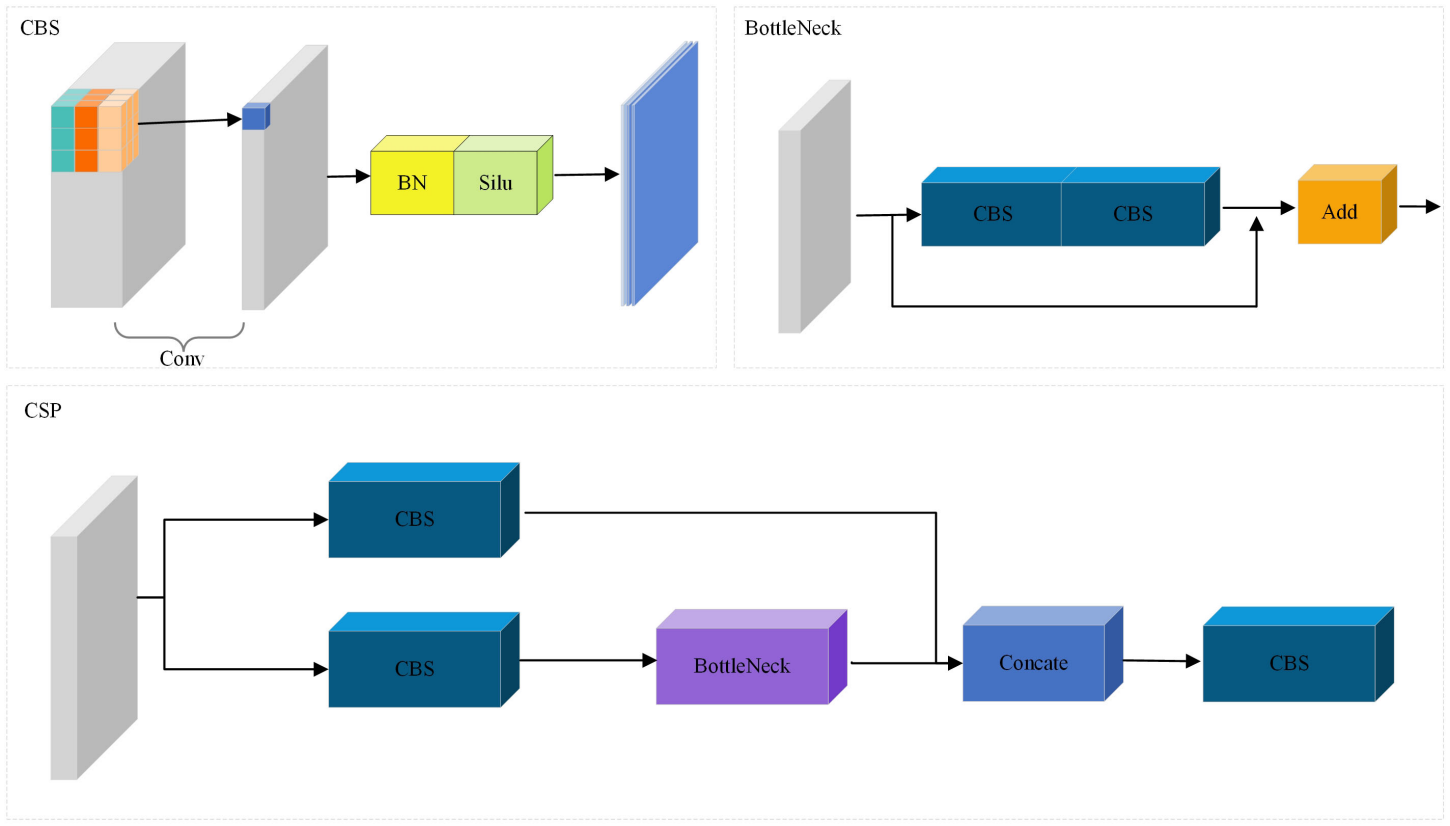
Illustration of CBS, BottleNeck and CSP.

As shown in **Figure 3**, the core idea of the CSP structure is to divide the input feature map into two branches. One branch is responsible for extracting low-level features, while the other branch is responsible for further processing these features to obtain high-level semantic information. Feature fusion and recombination are realized by introducing cross connection and partial connection. This design can improve the ability of feature representation and enable the network to better capture feature information at different scales.

**Figure 4:**
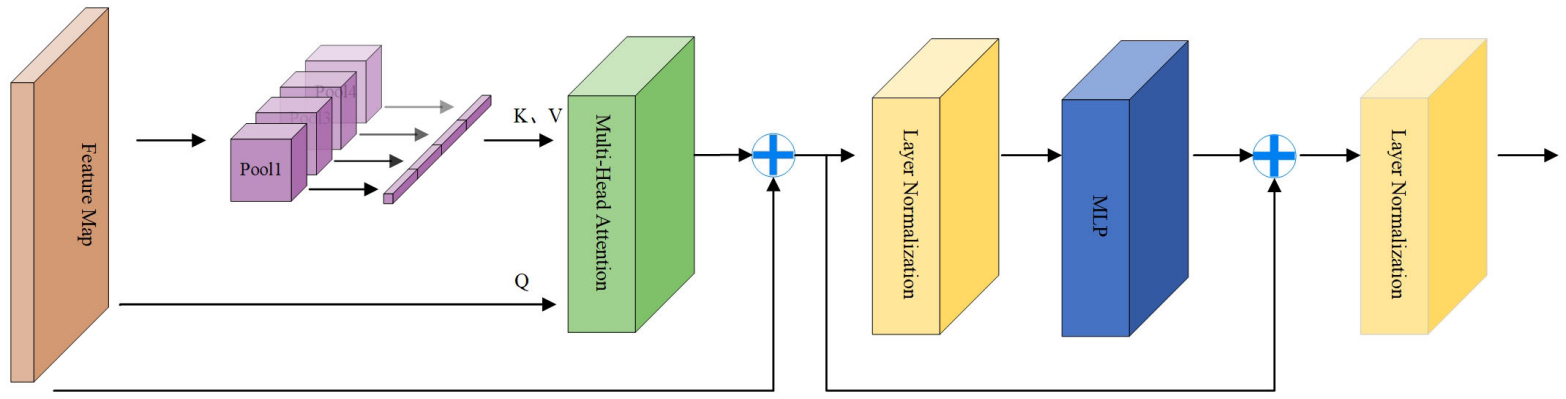
Architecture of the Multi-head Pyramid Pooling Transformer.

In addition, we also use the P2T structure to capture global context information. It introduces pyramid pooling in the multi-head self-attention module of vision Transformer. The benefits are reduced sequence length, reduced computation, and the ability to simultaneously learn strong contextual representations for better semantic features. The calculation process is shown in **Figure 4**. The input first passes through a pooling based multi head self-attention module, and its output adds residuals and performs normalization layer. Like traditional transformer blocks, a Feedforward Network (FFN) (Vaswani et al., 2017) is used for feature projection. Finally, apply residual connections and a normalization layer again.

Our backbone network combines CSP structure and P2T structure to form a hybrid network. Firstly, we use the CSP module to perform dimensionality reduction and local feature extraction on the input RGB image. The purpose of this method is to preserve key information while reducing subsequent computational complexity. Next, we use the P2T structure to capture global contextual information. In order to fully capture the multi-scale information of the target, we designed the feature extraction network as a pyramid structure. This is conducive to the generation of multi-scale feature representations and improves the performance and robustness of tasks.

#### 2.2.2 Feature fusion

Although the backbone network can extract multi-scale local features and global context information, the high-level features may lead to the loss and ambiguity of local information after deep convolution operation. High-level features focus on capturing more abstract and richer semantic features in the image. However, it loses some important details. On the contrary, low-level features are extracted from the shallow layer of the neural network and focus on capturing the details and local features of the input data. But it lacks high-level semantic information, and its feature representation capability is limited.

**Figure 5:**
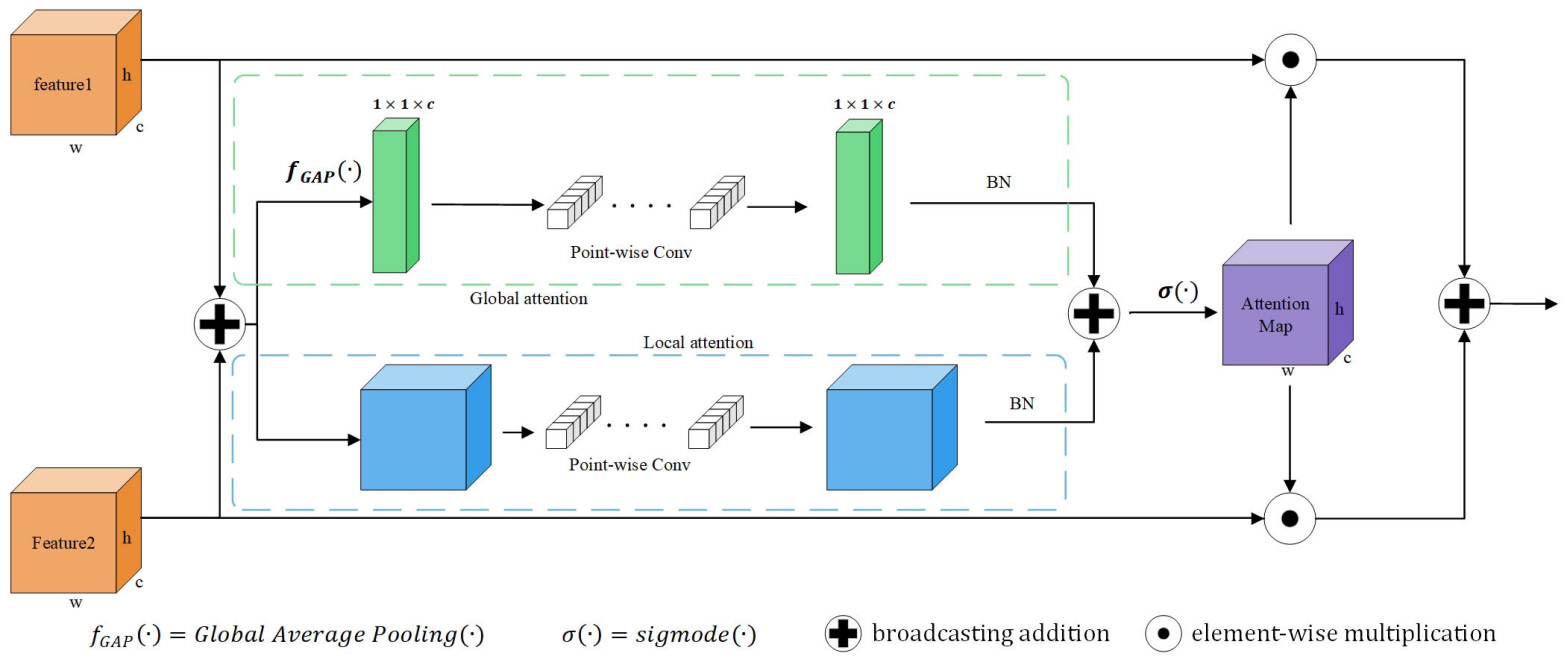
Illustration of the Attention Feature Fusion (AFF).

In order to fully integrate multi-scale features, we use an Attention Feature Fusion module (AFF) (Dai et al., 2021). This module uses two branches with different scales to extract channel attention for global features and channel attention for local features. As shown in **Figure 5**, the key idea is that channel attention can be achieved at multiple scales by varying the size of spatial pooling.

The purpose of AFF module is to comprehensively use global and local feature information to enhance the ability of feature representation. Global attention helps the model focus on the semantic content of the overall image, while channel attention helps the model adaptively learn the importance of different channels, so as to further integrate the information of different features. In this way, the local features and global context information in the image can be better fused, so as to improve the performance and robustness of the model.

#### 2.2.3 Loss function

Most of the existing methods are based on the assumption of pixel independence and use pixel-wise Euclidean loss to train the network. It is defined as follows:


$$L_{den}=\frac{1}{N}\sum_{i=1}^{N}{\left\|P\left(x_{i}\right)-G_{i}\right\|}^{2}$$

Where $P\left(x_{i}\right)$ is the predicted density map of the i th input image, $G_{i}$ is the true density map of the i th input image, $x_{i}$ represents the i th input image, and N is the number of input images.

In addition to the pixel-wise loss function, we add a local correlation loss to the density map to improve the quality of the predicted density map. The SSIM index is used to measure the local pattern consistency between the estimated density map and the ground truth. From three local statistics, namely mean, variance and covariance, the similarity between two images is calculated (Cao et al., 2018). The local statistics are computed by:


$$\mu_{x}=\frac{1}{M}\sum_{i=1}^{M}x_{i}$$

$$\sigma_{x}^{2}=\frac{1}{M-1}\sum_{i=1}^{M}{\left(x_{i}-\mu_{x}\right)}^{2}$$

$$\sigma_{xy}=\frac{1}{M-1}\sum_{i=1}^{M}\left(x_{i}-\mu_{x}\right)\left(y_{i}-\mu_{y}\right)$$

Where $\mu_{x}$ and $\sigma_{x}^{2}$ are the local mean and variance estimates of image x, M represents the number of pixels of image x, and $\sigma_{xy}$ is the local covariance estimate, so the SSIM index is calculated point by point as follows:


$$SSIM\left(x,y\right)=\frac{\left(2\mu_{x}\mu_{y}+C_{1}\right)\left(2\sigma_{xy}+C_{2}\right)}{\left(\mu_{x}^{2}+\mu_{y}^{2}+C_{1}\right)\left(\sigma_{x}^{2}+\sigma_{y}^{2}+C_{2}\right)}$$

Where $C_{1}$ , $C_{2}$ are minimal constants, to avoid division by zero and set as (Wang et al., 2004). The SSIM value ranges from -1 to 1 and takes the value 1 when two images are identical. Therefore, the local consistency loss is as follows.


$$L_{C}=1-SSIM\left(x,y\right)$$

$L_{C}$ is the local pattern consistency loss, which measures the local pattern difference between the estimation results and the ground truth.

In addition to computing the predictive density map loss, we also introduce an attention map loss function, which is a binary class entropy defined as follows.


$$L_{att}=-\frac{1}{N}\sum_{i=1}^{N}\left(A_{i}^{GT}\text{ log }\left(P_{i}\right)+\left(1-A_{i}^{GT}\right)\text{ log }\left(1-P_{i}\right)\right)$$

Where $A_{i}^{GT}$ is the attention map groundtruth and $P_{i}$ is the probability that each pixel in the predicted attention map is activated by the sigmoid function.

For high-density wheat ear images in complex backgrounds. Euclidean loss is used to train counting loss, while SSIM is used to train density map similarity loss. By combining these two loss functions, the aim is to improve the counting accuracy of high-density wheat ear images in complex backgrounds. The entire network is trained using the following unified loss function:


$$\text{L}={\text{L}}_{\text{den}}+{\text{L}}_{\text{c}}+\text{α}{\text{L}}_{\text{att}}$$

Where α is a weighted weight, which is set to 0.1 (Zhu et al., 2019) in the experiments. We exploit this combined loss for end-to-end training.

## 3 Experiment and result

This section presents the metrics used for the experimental evaluation, the training hyperparameters, and the final results. All experiments were trained on a computer equipped with a 24GB Geforce RTX 3090 graphics card. The deep learning framework used in the code is Pytorch 1.12 and the programming language is python 3.9. Adam (Adaptive Gradient Descent) method is used to optimize the learning rate during training. The initial learning rate is set to 0.0001. This configuration allows model training and evaluation to be performed efficiently.

### 3.1 Model performance evaluation

The performance of the model in the prediction and counting accuracy of Macintosh was evaluated using the mean absolute error (MAE), root mean square error (RMSE) and mean absolute percentage error (MAPE) as indicators. MAE and RMSE are commonly used in previous density estimation studies to measure the error between the predicted value and the actual value. MAPE is a measure of relative error, using absolute values to avoid bias between positive and negative errors, and is one of the most commonly used metrics for evaluating prediction performance. They can be expressed as:


$$MAE=\frac{1}{N}\sum_{i=1}^{N}\left|P_{i}-G_{i}\right|$$

$$RMSE=\sqrt{\frac{1}{N}\sum_{i=1}^{N}{\left|P_{i}-G_{i}\right|}^{2}}$$

$$MAPE=\frac{100\%}{N}\sum_{i=1}^{N}\left|\frac{P_{i}-G_{i}}{G_{i}}\right|$$

Where N is the number of test images, $\bar{P}$ is the average value of the predicted value, $P_{i}$ and $G_{i}$ are the predicted number and the real value in the i -th image respectively. RMSE is used to measure the average error between the predicted value and the real value of the model. The smaller the RMSE, the closer the prediction of the model to the real value. MAE is used to measure the average absolute error between the predicted value and the real value of the model. The smaller the MAE, the more accurate the model prediction and the higher the counting accuracy of the model. By calculating these indicators, we can evaluate the accuracy of the model in predicting the number of wheats ears and compare it with the actual value manually marked to measure the performance and accuracy of the model.

### 3.2 Ablation experiments

In this study, we used ablation experiments to explore the effects of P2T, CSP and SSIM loss on the model. As shown in **Table 2**, where V1 represents the model built only with CSP, V2 represents the integration of P2T module on the basis of V1, similarly, V3 represents the addition of AFF multi-scale fusion on the basis of V1, and CTHNet represents the final algorithm proposed in this paper, V4 represents the result obtained by training the model without using SSIM loss. The test results show that the integrated P2T and CSP are sufficient to ensure acceptable accuracy (MAE=5.61, RMSE=6.77). Moreover, the addition of AFF further integrates local features and global context information and leads to more accurate detection accuracy (MAE=5.21, RMSE=5.27), compared with using CSP alone. SSIM loss is used to compare the similarity between real density maps and predicted density maps. By adding SSIM loss, the model’s counting performance can be effectively improved. **Table 2** shows a decrease of 0.18 in MAE and 1.48 in RMSE. These findings confirm that the model effectively improves the counting accuracy of the model by fusing global context information and local features.

**Table 2:** Performance metrics of CTHNet before and after improvement on the WEDD.

Model	CSP	P2T	AFF	SSIM	MAE	RMSE
V1	√	–	–	√	5.67	7.26
V2	√	√	–	√	5.61	6.77
V3	√	–	√	√	5.68	7.95
V4	√	√	√	–	5.39	6.75
CTHNet	√	√	√	√	5.21	5.27

**Figure 6:**
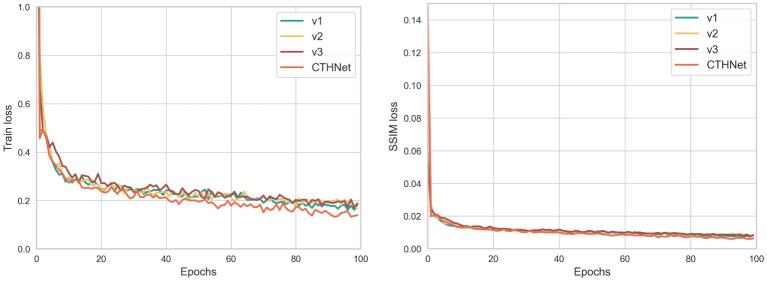
Results from the training process: Train loss and SSIM loss.

**Figure 7:**
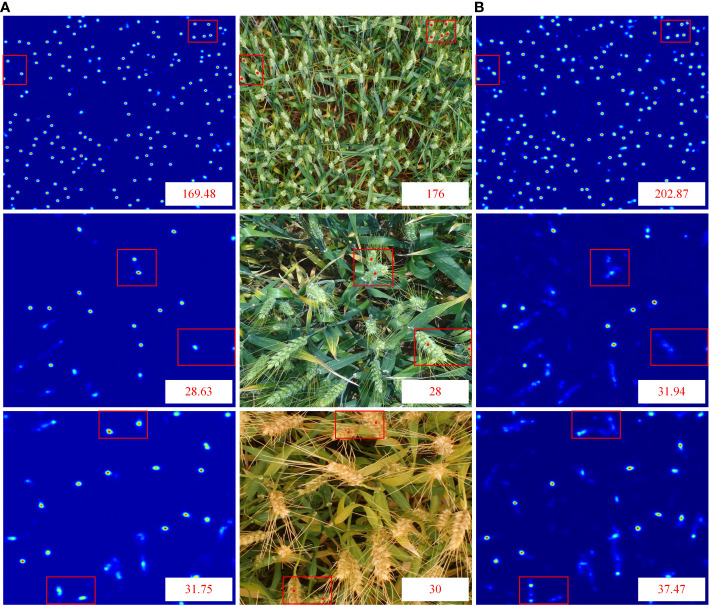
Comparison of density maps: **(A)** Density maps generated by combining local and global features **(B)** Density maps generated based on local features.

We showed the change in loss curves to provide a more intelligible representation of the loss change. **Figure 6** depicts the variations in training loss and SSIM loss values for each improved version. It can be observed from **Figure 6** that the loss value decreases rapidly at the beginning of training, and with the increase of training epochs, the training loss gradually decreases and fluctuates near a critical value. From **Figure 7**, it can be seen that the density map generated by integrating local and global features is clearer and more pronounced than the density map generated solely based on local features, especially in the edge and high-density areas. Notably, the loss function of the final algorithm proposed in this paper still continues to decrease over a range of 40 to 100 epochs, suggesting that the model has a good capacity for learning.

### 3.3 Comparison experiments

In recent years, density regression algorithms have progressed quickly and been used in a variety of counting applications. We contrast our method with various density counting methods, such as MCNN (Zhang et al., 2016), SANet (Cao et al., 2018), SCAR (Gao et al., 2019), PSNet (Cheng et al., 2020), TasseLnetv2+ (Lu and Cao, 2020) and CCTrans (Tian et al., 2021). We evaluated the counting performance of the proposed model on two public wheat ear detection datasets GWHD and WEDD (**Table 3**).

**Table 3:** Compared with other density estimation methods on the GWHD and WEDD dataset.

Model	GWHD			WEDD		
	MAE	RMSE	MAPE	MAE	RMSE	MAPE
MCNN	8.49	17.61	27.65	10.69	14.29	7.79
SANet	4.80	11.28	13.72	11.88	13.80	8.78
SCAR	4.48	8.03	15.63	6.38	8.59	4.77
TasseLnetv2+	6.44	11.97	20.50	7.93	9.97	5.97
CCTrans	5.10	11.07	15.01	6.91	8.45	5.26
PSNet	4.62	8.64	15.64	6.29	7.86	4.77
CTHNet	3.40	5.75	12.47	5.21	5.27	3.95

**Figure 8:**
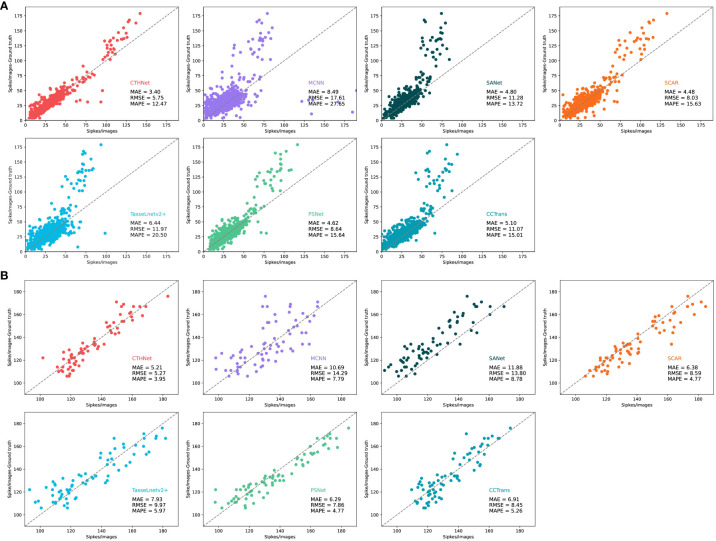
Comparison of the number of real ears annotated on the GWHD test dataset **(A)** and the WEDD test dataset **(B)** with that estimated by MCNN, SANet, SCAR, TassLnetv2+, PSNet, CCTrans and CTHNet.

**Figure 9:**
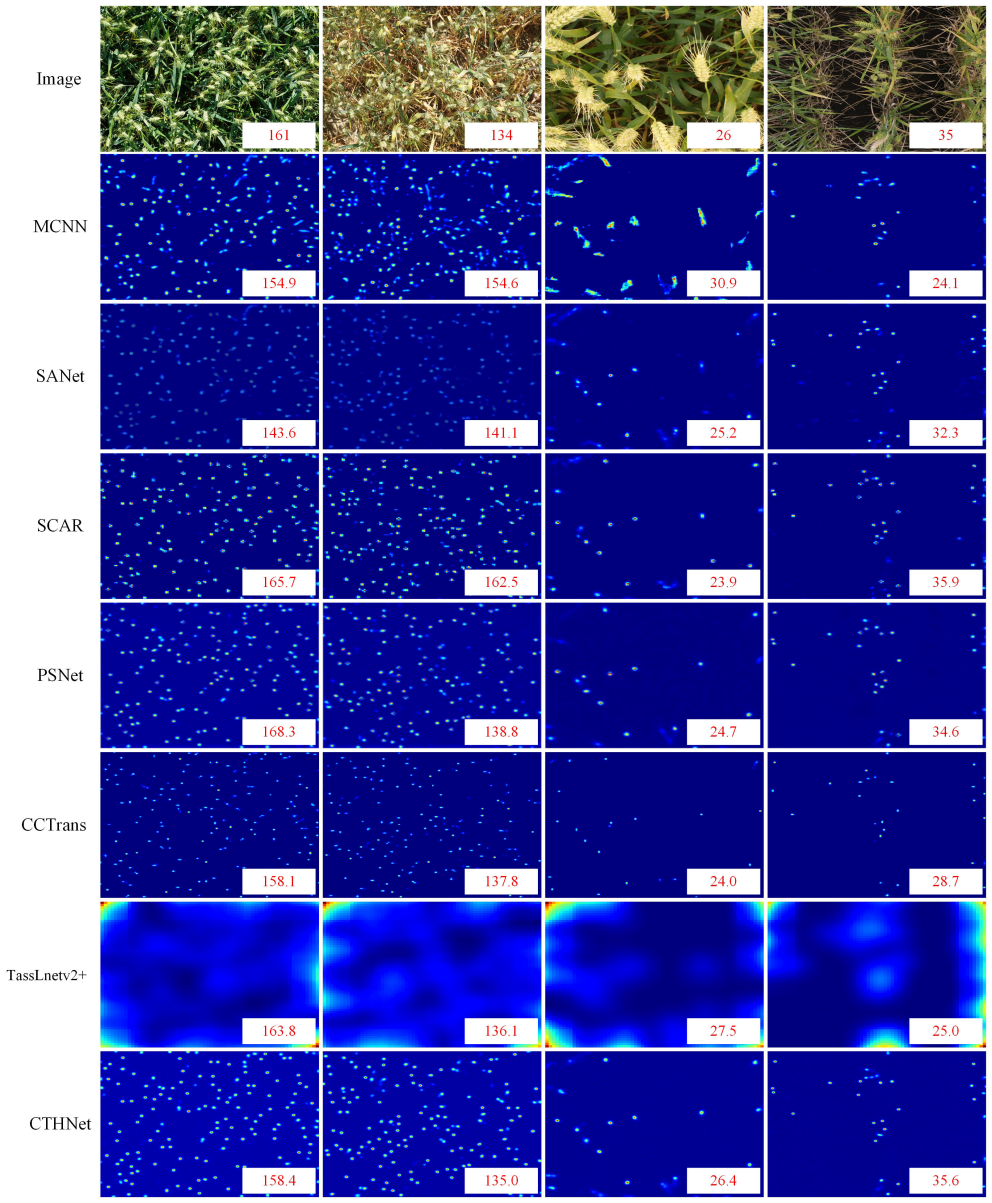
Comparison of predicted density maps generated by different models on images.

We perform visual analysis on two datasets to show the counting performance of the proposed method more intuitively and scientifically. We use the test images of GWHD and WEDD datasets to compare the number of true peak annotations of various counting methods (**Figure 8**). We also collected and compared the predicted density maps generated by different counting models (**Figure 9**).

**Table 3**, show that hybrid network counting performs better than other counting methods based on density estimation. Since the ear of wheat is a small target, its feature is easily contaminated during the extraction process by examples like background, which makes it challenging for the network to capture the discriminative information required for following tasks (Shi et al., 2023). CNN-based counting models MCNN, SANet and TasseLnetv2+ learn high-dimensional features and reduce spatial redundancy through down-sampling operations, but this leads to the loss of representation for small objects, making accurate counting difficult. To overcome this problem, CCTrans uses a pyramid visual transformer backbone to capture global information and complete the counting task by combining low-level and high-level features. The experimental results show that CCTrans performs well in counting effect. In addition, SCAR and PSNet show better counting performance than MCNN and SANet by adding attention to enhance the extraction ability of small target features, but they fall short of our proposed model. This is because the convolution operation mainly extracts semantic features at high-level, and the potential features for small targets may be lost or compressed. The convolutional network also has some challenges in the recognition of small targets, especially when dealing with scenes with complex backgrounds. Convolutional operations and self-attention mechanisms are used to extract multi-scale local features and global context information. This lessens the detrimental effects of the convolution constraint by enabling the full utilization of the global context information. Meanwhile, a feature fusion module based on local and global attention is used to combine local features with global context information, improving the capability of feature representation.

In addition, we use dilated convolution to improve the density map’s receptive field, keeping the context and small item details of the wheat image. Compared with other wheat ear number models, the network model performs well in complex background (**Table 3**). Compared with SANet, MCNN, PSNet, SCAR, TasseLnetv2+ and CCtrans. MAE improves the performance on GWHD dataset by 6.67, 5.48, 1.08, 1.17, 2.04, 1.70. On the WEDD dataset, the performance is improved by 1.40, 5.09, 1.22, 1.08, 2.72, 1.70. By looking at the density plots of the predictions of different models (**Figure 9**). We find that the predictive density maps generated by the hybrid network can better approximate the ground truth than the other models.

On the test images of the two datasets, we compared the number of true peak annotations for various counting methods (**Figure 8**). It can be found that the performance of the model on the GWHD dataset is better than the WEDD dataset. This is mainly because the wheat density in WEDD dataset is much higher than GWHD, and the occlusion between wheat ears and background noise are more likely to cause confusion interference, which increases the difficulty of feature extraction and counting. However, on the high-density WEDD dataset, the hybrid model continues to outperform the other models in terms of counting ability, indicating that the hybrid network design can successfully address the issues of misdetection and omission brought on by the overlap of wheat ear counts and the complexity of the background. The proposed method not only improves the accuracy of the model, but also has wide applicability and is able to deal with wheat counting tasks in different scenarios. This is of great significance for improving the efficiency of agricultural production, reducing resource waste, and for farmland management and crop production.

### 3.4 Discussion

We create a hybrid network using convolution operation and self-attention mechanism to fully extract local features and global context information of wheat ear images. Experimental results on two public datasets show that the proposed method has good counting and generalization ability.

Firstly, SCAR and PSNet with the added attention mechanism outperform the convolution based MCNN, SANet and TasseLnetv2+ in counting performance. This is because the attention mechanism highlights the key features of the wheat ears and ignores unnecessary regions by assigning different weights to different parts of the feature map. However, since the context information in the image is ignored, SCAR and PSNet with the attention mechanism are not as good as the hybrid network proposed in this study in counting performance. CCTrans utilizes the pyramid vision transformer as a backbone, making it easy to extract global context information. However, the counting results of CCTrans in GWHD and WEDD are still lower than the model proposed in this paper. In contrast, by combining the convolutional operation with the self-attention mechanism and adding the attention feature fusion to the hybrid model, the global context information and local details of the image can be effectively fused. By fusing these two kinds of information, the relationship between various spatial information can be strengthened, thus improving the recognition ability of wheat ears on the feature map.

In this study, we realized that there are some differences in features between wheat images and dense crowd images. The shape of a wheat ear is usually rectangular, while the shape of a human head is round. Therefore, the traditional annotation methods and density map generation methods for dense crowd counting tasks cannot describe the characteristics of wheat ears well. To solve this problem, we plan to design a suitable labeling method for wheat ears to accurately describe the shape and location of wheat ears. For example, we can use lines to describe the shape of the wheat ear or make point annotations on the head of the wheat ear to describe the position of the wheat ear. With this more refined labeling method, the characteristics of the wheat ear can be better captured, including the shape characteristics and location characteristics of the wheat ear.

Additionally, the phenotypic characteristics of a single wheat ear, including the spike length, spike width, and grain number, are significant indicators of the quality and growth of the crop. The traditional way of gathering phenotypic information on wheat ears includes manual measurement and counting, which is time-consuming and labor-intensive and significantly slows down wheat ear research. We will implement the batch extraction of phenotypic data from single wheat ears using the above-mentioned study methods and data combined with an example segmentation algorithm. We will next propose a scheme for extracting phenotypic information of wheat ears in a complex background in the field. Through this program, the staff can collect wheat phenotype data in the field in real time, saving data collection time and improving research efficiency, and provides strong support for agricultural breeding and phenotyping work.

## 4 Conclusion

In this study, we propose a hybrid network model that combines convolutional operations and a self-attention mechanism. This model is used to generate high-quality wheat ear density maps for accurately calculating the number of wheat ears in complex background images. The results demonstrate a significant improvement in the wheat ear counting task achieved by the hybrid network. By fusing local features of wheat ears and global context information, the edge and small target information in the image can be effectively preserved, and the counting performance is improved.

The hybrid network has good feature representation ability and can meet the requirements of wheat ear number under the conditions of occlusion and overlap. It provides a reliable estimate of wheat yield and provides strong support for agricultural production. In the future research, we will focus on the design of labeling method and density map generation method for wheat counting task, in order to further improve the accuracy and performance of hybrid network. We will also develop precision agriculture applications that utilize unmanned aerial vehicle (UAV) to collect wheat ear image data at different times, varieties and planting densities, further validating the performance of the model and improving its generalization ability. This will provide real-time and accurate information for agricultural production, help farmers make scientific decisions, and improve crop management and yield.

## Data availability statement

Publicly available datasets were analyzed in this study. This data can be found here: http://www.global-wheat.com/ https://github.com/simonMadec.

## Author contributions

QH: Conceptualization, Formal analysis, Investigation, Methodology, Validation, Visualization, Writing – review & editing. WL: Conceptualization, Formal analysis, Investigation, Methodology, Software, Validation, Visualization, Writing – original draft. YZ: Software, Writing – review & editing. TR: Software, Writing – review & editing. CS: Supervision, Writing – review & editing. ZL: Supervision, Writing – review & editing. YY: Supervision, Writing – review & editing. RD: Supervision, Writing – review & editing. JQ: Supervision, Writing – review & editing. CT: Supervision, Writing – review & editing.
